# In Silico Design of Dual-Binding Site Anti-Cholinesterase Phytochemical Heterodimers as Treatment Options for Alzheimer’s Disease

**DOI:** 10.3390/cimb44010012

**Published:** 2021-12-29

**Authors:** Hafsa Amat-ur-Rasool, Mehboob Ahmed, Shahida Hasnain, Abrar Ahmed, Wayne Grant Carter

**Affiliations:** 1 School of Medicine, Royal Derby Hospital Centre, University of Nottingham, Derby DE22 3DT, UK; hafsa.phd.mmg@pu.edu.pk; 2 Department of Microbiology and Molecular Genetics, University of the Punjab, Lahore 54590, Pakistan; mehboob.mmg@pu.edu.pk (M.A.); shahida.mmg@pu.edu.pk (S.H.); 3 Faculty of Pharmacy, Punjab University College of Pharmacy, University of the Punjab, Lahore 54590, Pakistan; abrar.pharmacy@pu.edu.pk

**Keywords:** acetylcholinesterase, Alzheimer’s disease, butyrylcholinesterase, dual-binding site cholinesterase inhibitors, heterodimers, phytochemicals

## Abstract

The number of patients with neurodegenerative diseases, particularly Alzheimer’s disease (AD), continues to grow yearly. Cholinesterase inhibitors (ChEIs) represent the first-line symptomatic drug treatment for mild-to-moderate AD; however, there is an unmet need to produce ChEIs with improved efficacy and reduced side effects. Herein, phytochemicals with reported anti-acetylcholinesterase (AChE) activity were ranked in silico for their anti-AChE potential. Ligands with a similar or higher binding affinity to AChE than galantamine were then selected for the design of novel dual-binding site heterodimeric drugs. In silico molecular docking of heterodimers with the target enzymes, AChE and butyrylcholinesterase (BuChE), were performed, and anti-cholinesterase binding affinities were compared with donepezil. Drug-likeliness properties and toxicity of the heterodimers were assessed using the SwissADME and ProTox-II webservers. Nine phytochemicals displayed similar or higher binding affinities to AChE than galantamine: sanguinarine > huperzine A > chelerythrine > yohimbine > berberine > berberastine > naringenin > akuammicine > carvone. Eleven heterodimeric ligands were designed with phytochemicals separated by four- or five-carbon alkyl-linkers. All heterodimers were theoretically potent AChE and BuChE dual-binding site inhibitors, with the highest affinity achieved with huperzine-4C-naringenin, which displayed 34% and 26% improved affinity to AChE and BuChE, respectively, then the potent ChEI drug, donepezil. Computational pharmacokinetic and pharmacodynamic screening suggested that phytochemical heterodimers would display useful gastrointestinal absorption and with relatively low predicted toxicity. Collectively, the present study suggests that phytochemicals could be garnered for the provision of novel ChEIs with enhanced drug efficacy and low toxicity.

## 1. Introduction

The healthcare burden from neurodegenerative diseases has increased monotonically due to increased longevity and a relatively high proportion of the population that are geriatrics. Current estimates have suggested that at least 50 million people are living with dementia, with an annual societal and economic healthcare cost of over $800 billion [1]. The most common form of dementia is Alzheimer’s disease (AD), a progressive neurodegenerative disease typified by symptoms of cognitive decline including episodic memory loss and confusion, as well as visuospatial problems and behavioral and psychiatric changes [2].

The current first-line symptomatic drug treatment for AD is cholinesterase inhibitors (ChEIs) and memantine, an NMDA receptor antagonist. The US food and drug administration (FDA) approved ChEIs, donepezil, rivastigmine, and galantamine provide pharmacotherapy for the treatment of mild-to-moderate AD [3,4]. The mode of action of ChEIs is via transient binding and inhibition of acetylcholinesterase (AChE) within the central nervous system (CNS). This drug treatment strategy is to prolong the activity of the neurotransmitter acetylcholine and thereby sustain cholinergic innervations. This is particularly pertinent for the cholinergic projections from the basal forebrain to the frontal cortex and to the hippocampus, for which a decline in functionality of the latter is linked to the memory deficits associated with AD progression [2,5].

When AChE activity is absent, butyrylcholinesterase (BuChE) can substitute for AChE to limit ACh signaling [6]. Studies have reported increased BuChE activity in AD [7,8,9], perhaps to compensate for reduced AChE activity. Therefore, the targeted inhibition of BuChE, or moreover, the utilization of drugs capable of dual inhibition of AChE and BuChE such as rivastigmine, may prove better agents for sustaining ACh signaling [10,11].

There are limitations with the current pharmacotherapy approach, including elicitation of adverse drug reactions (ADRs) and restricted drug efficacy and patient responsiveness, such that the cost-effectiveness of ChEI treatment has been questioned [12]. However, although ChEIs are not able to completely arrest disease progression, their chronic use results in a modest but persistent benefit to cognition with a reduced risk of mortality [13]. Hence, therapeutic benefits have driven the search for ChEIs that display similar or improved clinical efficacy to the currently employed ChEIs, but with reduced ADRs.

Plants used in traditional herbal and folkloric medicine liberate phytochemicals that bind and inhibit AChE, and they thereby provide alternative sources of natural rather than synthetic ChEIs [14,15,16,17,18]. Phytochemicals from traditional medicine, such as huperzine A, have proven beneficial to cognition, are well-tolerated, and has AChE inhibitory efficacy superlative to galantamine (0.08 µM compared to 2.0 µM, respectively) [19,20,21]. Additionally, herbal remedies have a perceived lower toxicity than synthetic drugs and are generally regarded as safe (GRAS) due to their natural origin [22].

The active site of AChE possesses an esteratic (catalytic) subsite and a peripheral subsite. The peripheral anionic site (PAS) has a gorge-like entrance with several aromatic amino acids that mediate ACh trapping and lead down to the catalytic site (CS), at which a catalytic triad of Ser200, Glu327, and His440 mediate substrate hydrolysis [23,24,25]. ChEIs, such as donepezil, can inhibit AChE activity via transitory binding to the PAS and/or the catalytic site of the enzyme [25,26]. Potential control of AChE activity via dual inhibitory binding site occupancy has led to the generation of hybrid ligands separated by suitable linkers or spacers. Homo- or heterodimers exhibit potent enzyme inhibition efficacy, as assessed using computational and pre-clinical studies [27,28,29,30,31,32,33].

ChEIs able to bind at the PAS may also display AD-modifying non-classical and non-cholinergic properties, including a reduction of aggregation of amyloid-beta (Aβ), one of the pathological hallmarks of AD [27,32,34,35,36].

Rational synthesis of dual-binding site ligands has included drug candidates with inhibitory potency against both AChE and BuChE to potentially further support sustained ACh levels in AD [30,31,34]. Furthermore, to potentially reduce ADRs, dual-binding site ChEIs have been designed with phytochemical building blocks [29,37,38].

Herein, phytochemicals with documented AChE inhibitory activity were ranked, in silico, for their potency as inhibitors of AChE. Compounds with the most potent inhibitory effects were then selected as monomers, from which novel heterodimeric dual-binding site anticholinesterase drugs were designed. These drugs were subsequently screened for their potency as ChEIs, and an assessment of their drug-likeliness and toxicity was undertaken computationally.

## 2. Materials and Methods

### 2.1. Screening and Molecular Docking of Phytochemicals

A search of plants and phytochemicals with reported anticholinesterase activity was conducted using Dr Duke’s Phytochemical and Ethnobotanical Databases, US Department of Agriculture (https://phytochem.nal.usda.gov/phytochem/search, accessed on 25 September 2021) (Supplementary Table S1) [39]. The 3D structures of these compounds were downloaded from the NCBI-PubChem database, and the 3D structure of the target AChE enzyme (PDB ID: 4EY5) was downloaded from the RCSB-Protein Data Bank (PDB). The AChE enzyme structure was cleaned using PyMOL v2.1 software and used for molecular docking studies with phytochemical ligands using AutoDock Vina tools (http://vina.scripps.edu/, accessed on 25 September 2021). Ligands were ranked according to the highest binding affinity to the target AChE enzyme.

### 2.2. Design and Molecular Docking of Heterodimeric Ligands

Ligands that displayed a higher binding affinity to AChE than galantamine were selected for the design of novel dual-binding site heterodimeric drugs. ChemDraw Ultra 8.0 software [40] was used to design compounds with two phytochemical units attached with a carbon linker of suitable length. Heterodimeric drugs were designed on the premise that, even with an alkyl linker, the molecular weight would not exceed 600 Da but would still fill the AChE catalytic groove. Target AChE and BuChE enzyme structures (PDB ID: 2CMF and PDB ID: 6I0C, respectively) were retrieved from the PDB and cleaned using PyMOL v2.1 software. Molecular docking of the newly designed heterodimers or donepezil with each target enzyme were performed using AutoDock Vina tools. Docking results for the new heterodimers were analyzed by superimposing them with the original heterodimer ligands for the target enzymes, and these were then ranked according to their binding affinities. Docking simulation images were generated using PyMOL v2.1 software, with the lengths of hydrogen bonds between ligands and the target enzymes measured in Å.

### 2.3. Pharmacokinetic Predictions

The screened phytochemicals and newly designed ligands were subjected to drug-likeliness studies using SwissADME (http://www.swissadme.ch/index.php, accessed on 28 November 2021). This program provided physicochemical properties and ADME (absorption, distribution, metabolism, and excretion) parameters associated with the pharmacokinetics of each compound. Data calculated included the number of rotatable bonds, hydrogen bond acceptors, hydrogen bond donors, log P_o/w_ values as a measure of lipophilicity, and gastrointestinal (GI) absorption [41].

### 2.4. Pharmacodynamic Predictions

The phytochemicals and heterodimeric drug candidates were subjected to in silico pharmacodynamic studies to determine their potential cytotoxicity using the online webserver ProTox-II (https://tox-new.charite.de/protox_II/, accessed on 28 November 2021) [42]. ProTox-II is a virtual lab for the predictive toxicity of small molecules, a process that can reduce the need for subsequent drug (in vivo) testing in animals. The ProTox-II server was used to determine predicted acute oral toxicity of the phytochemical compounds in rodents based upon 2D structure similarities with 38,000 compounds (provided by the software) and their associated LD_50_ values. Additionally, hepatotoxicity was predicted via ProTox-II based on machine learning models.

### 2.5. Molecular Dynamics Simulations

The stability and interaction of AChE and BuChE targets with the most suitable ligands were determined by using the Maestro-Desmond v12.3 Schrödinger software package [43]. The docking complex was placed in an orthorhombic box and water molecules were added. The charge of each system was neutralized by adding Na^+^ or Cl^−^ ions, and then the system was minimized and pre-equilibrated. Each molecular dynamic simulation was run for a time of 50 ns using a normal pressure and temperature (NPT) ensemble of 300 K temperature and 1.013 bars pressure. Default settings were used to relax the system. Protein-ligand root mean square deviation (RMSD) and the amino acid residues involved in protein-ligand contact were analyzed.

## 3. Results

### 3.1. Screening of Anti-AChE Phytochemical Ligands

The human AChE enzyme (PDB ID: 4EY5) was utilized as a virtual target to rank phytochemicals according to their binding affinities. The phytochemical drug galantamine was used as a benchmark, and the ligands that were more potent than galantamine were selected for further analyses. The phytochemicals sanguinarine, huperzine A, chelerythrine, yohimbine, berberine, berberastine, naringenin and akuammicine displayed more potent AChE binding affinities (more negative kcal/mol values) than galantamine, with carvone identical to galantamine at −7.7 kcal/mol (Table 1).

**Figure 1:**
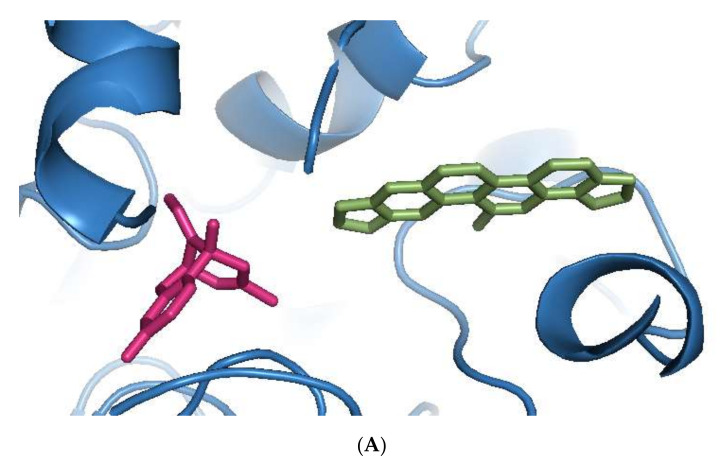

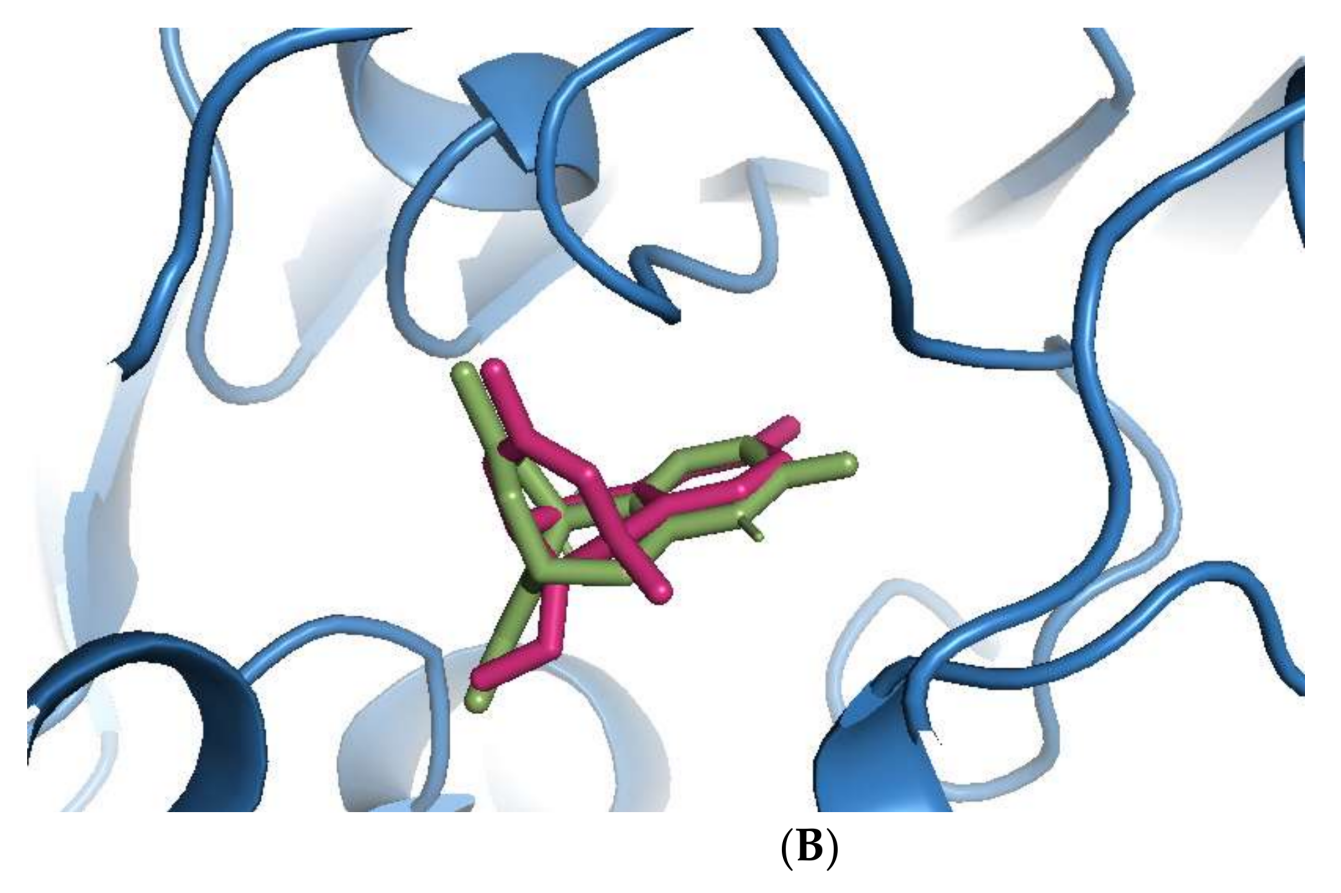
Binding of phytochemicals at different sub-sites of the AChE active site. (**A**) Superimposition of docked sanguinarine (green-colored sticks) and pre-bound ligand huperzine A (pink-colored sticks) to the active site in the human AChE crystal structure (PDB ID: 4EY5). (**B**) Superimposition of docked huperzine A (green-colored sticks) and pre-bound ligand huperzine A (pink-colored sticks) in the human AChE crystal structure (PDB ID: 4EY5).

Sanguinarine was computationally the most potent anti-AChE ligand, while huperzine A was second-best, with a 35% and 29% increase of binding affinity to that of galantamine, respectively. However, the binding positions of both ligands were different. Sanguinarine tended to bind to the PAS, while huperzine A inhibited AChE by binding to the deep CS (Figure 1A). The binding of both ligands was confirmed by their superimposition to huperzine A bound as a co-crystal structure to the human AChE enzyme [44] (Figure 1B). This indicates that AChE binding and inhibition could be achieved by either blocking the PAS or CS and, moreover, that two small drug molecules could be accommodated simultaneously at the AChE active site.

### 3.2. Molecular Docking of Heterodimeric Ligands

Since two phytochemical drugs could be accommodated within the active site of AChE, a single heterodimeric drug is feasible with a suitable linker region. However, in order to traverse the blood-brain barrier and exert CNS effects, drug molecular weight is usually restricted to a threshold of 600 Da [45]. Therefore, to design new heterodimeric combinations of the phytochemicals, a molecular weight cut-off of 600 Da was used. This resulted in 11 newly designed heterodimeric compounds that were then subjected to molecular docking to assess their potential binding to AChE (PDB ID: 2CMF) and BuChE (PDB ID: 6I0C) and to determine their respective binding affinities (Table 2). All the newly designed heterodimers had a higher binding affinity for AChE than BuChE and had higher values than those generated for donepezil (−10.5 kcal/mol and −9.8 kcal/mol for AChE and BuChE, respectively), the potent FDA approved ChEI drug.

Huperzine A, when linked with naringenin via a 4-carbon alkyl spacer (huperzine-4C-naringenin), was the most potent of the designed AChE inhibitors (at −14.1 kcal/mol) and likewise BuChE inhibitors (−12.3 kcal/mol). This novel heterodimer bound AChE with an improved affinity over that of donepezil or galantamine (34% and 83% higher, respectively), and it similarly displayed an improved affinity for BuChE over these FDA approved drugs by 26% and 41%, respectively.

**Figure 2:**
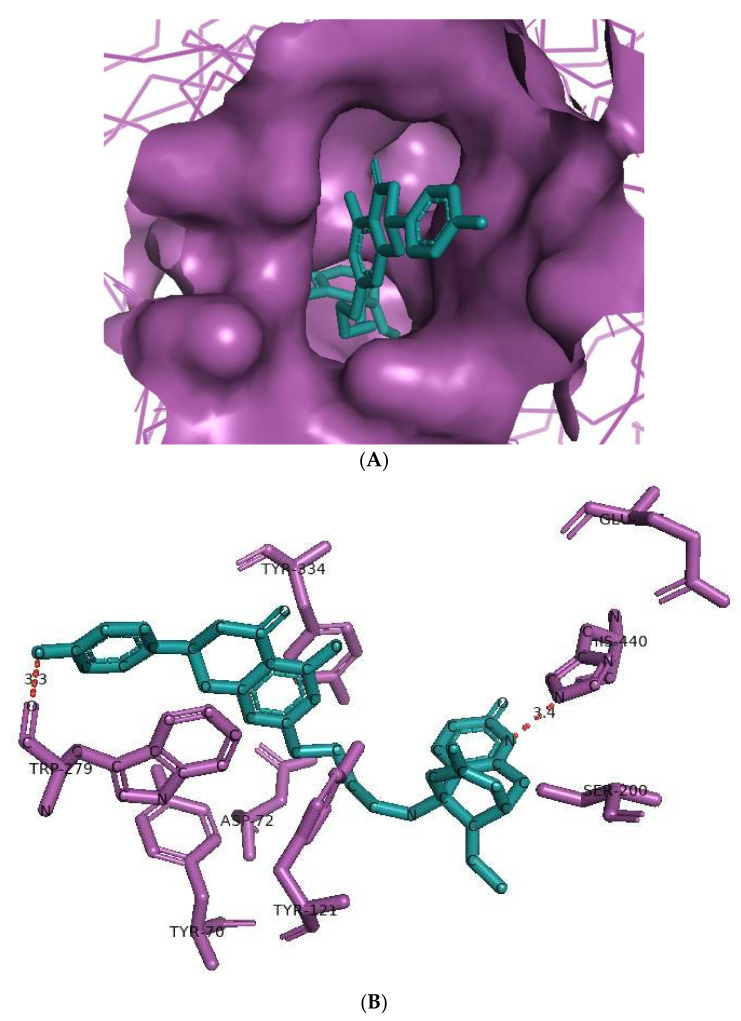

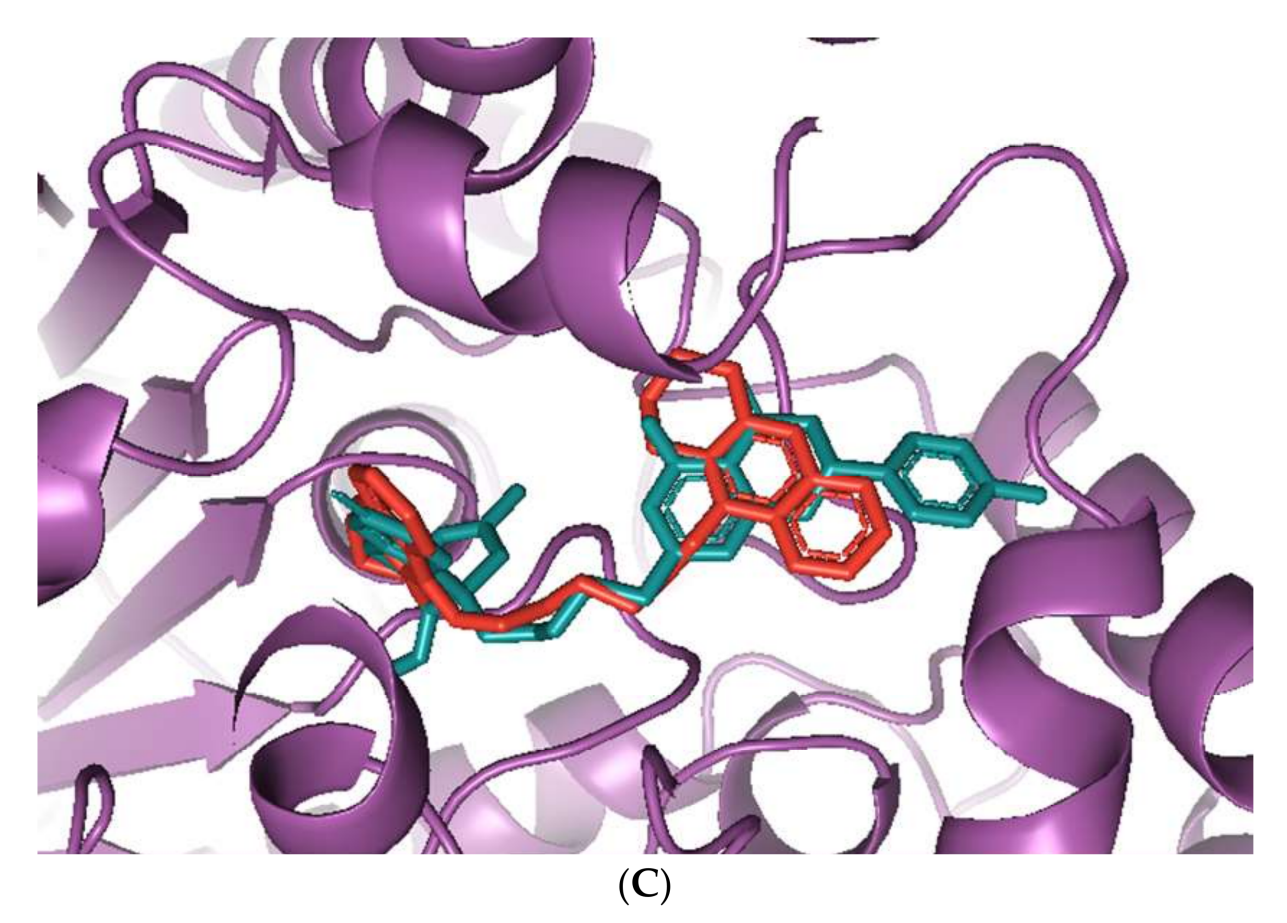
Docking models of huperzine-4C-naringenin (colored cyan) binding to the active site of AChE enzyme (colored purple). (**A**) Binding pose of huperzine-4C-naringenin looking down the catalytic gorge of AChE. (**B**) Ligand huperzine-4C-naringenin docked with the binding site residues of AChE. Hydrogen bonding is represented by red lines and the distance measured in Å. (**C**) Huperzine-4C-naringenin superimposed with a bis-tacrine dimer (colored red) pre-bound into the crystal structure of the AChE enzyme target (PDB ID: 2CMF).

The huperzine-4C-naringenin heterodimer could simultaneously bind the PAS and the CS of AChE to inhibit activity. The binding pose of huperzine-4C-naringenin along the active site gorge of the target AChE enzyme is shown in Figure 2A. The interaction of the ligand with amino acids of the active site is represented in Figure 2B. In addition to Van der Waals forces and ionic molecular interactions, ligand stability is facilitated by an O-O hydrogen bond of 3.3 Å length with Trp-279 of the PAS, as well as an N-O hydrogen bond of 3.4 Å length with one of the key residues of the catalytic triad, His-440, at the CS (Figure 2B). Superimposition of huperzine-4C-naringenin with a pre-bound bis-tacrine dimer in the co-crystal structure of AChE (PDB ID: 2CMF) [46] validates this binding (Figure 2C).

**Figure 3:**
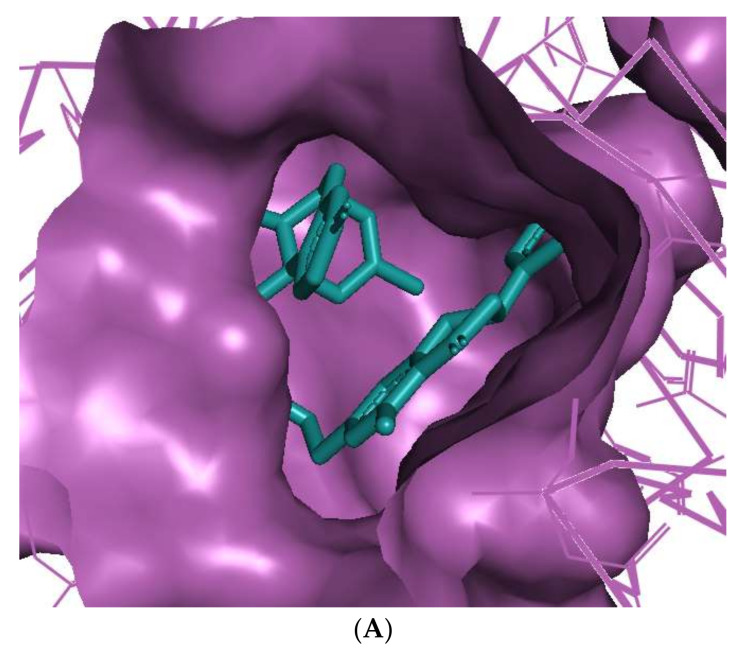

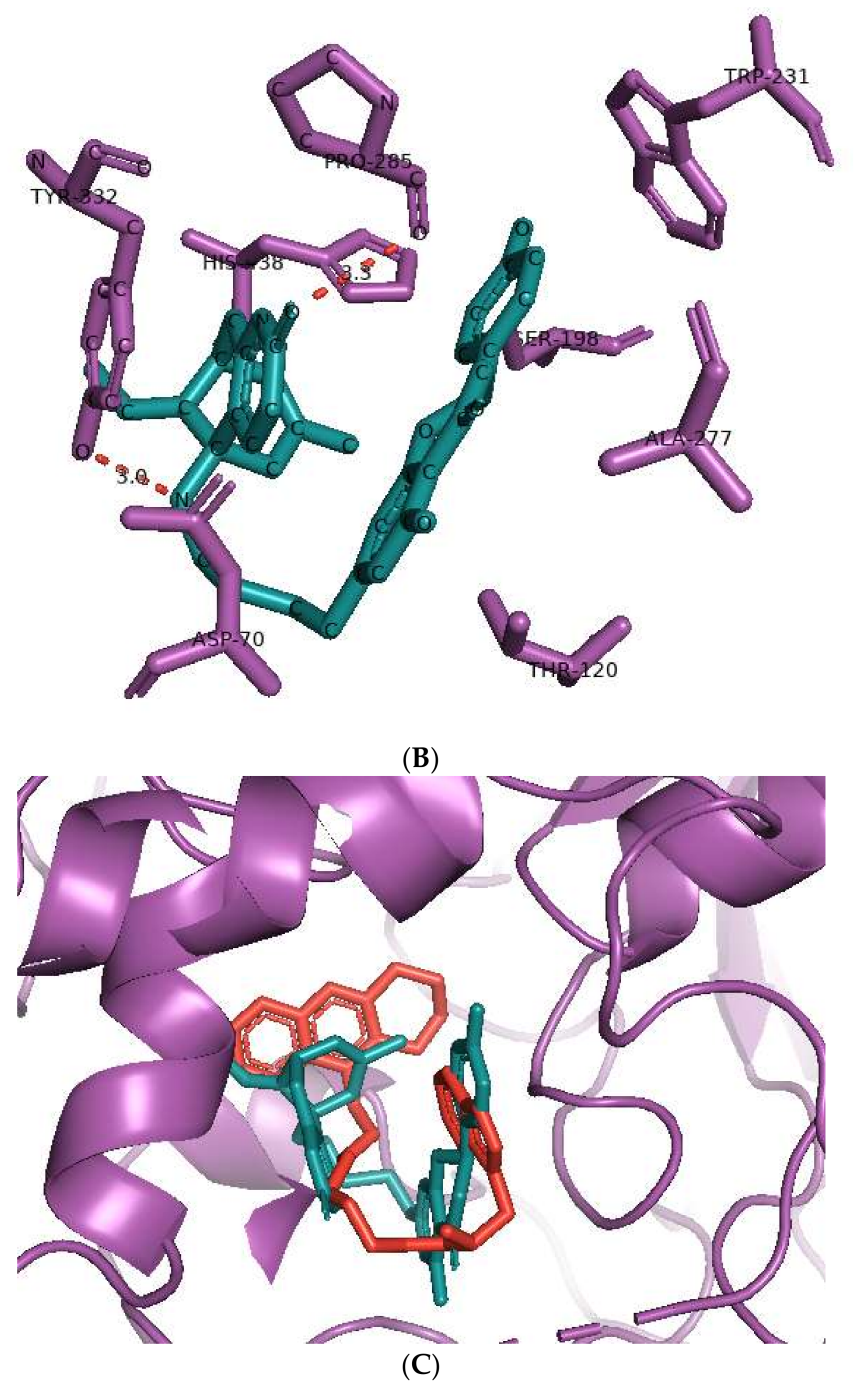
Docking models of huperzine-4C-naringenin (colored cyan) binding to the active site of BuChE enzyme (colored purple). (**A**) Binding pose of huperzine-4C-naringenin looking down the catalytic gorge of BuChE. (**B**) Ligand huperzine-4C-naringenin (cyan colored sticks) docked with binding site residues of BuChE. Hydrogen bonding is represented by red lines and the distance measured in Å. (**C**) Huperzine-4C-naringenin superimposed with a chlorotacrine-tryptophan heterodimer pre-bound with the crystal structure of the BuChE enzyme target (PDB ID: 6I0C).

The binding of huperzine-4C-naringenin within the active site of BuChE was also examined. Since the active site gorge of BuChE is wider than that of AChE, this ligand folds into a U-shape to block the active site (Figure 3A). The amino acid residues within BuChE active site that facilitate huperzine-4C-naringenin binding are shown in Figure 3B. Ligand stabilization is assisted by one N-O hydrogen bond with Tyr-332 of the PAS with a bond length of 3.0 Å, and one O-O hydrogen bond with Pro-285, with a bond length of 3.3 Å. Superimposing huperzine-4C-naringenin with that of a chlorotacrine-tryptophan heterodimer pre-bound into the co-crystal structure of BuChE (PDB ID: 6I0C) [47] validates this binding (Figure 3C).

### 3.3. ADME Prediction

The screened phytochemicals and newly designed drug candidates were subjected to drug pharmacokinetic analysis using the online prediction tool, Swiss-ADME. A summary of their predicted physicochemical properties with regards to molecular weight, number of rotatable bonds, number of hydrogen bond acceptors and hydrogen bond donors, octanol/water partition coefficient (log P_o/w_), and relative gastrointestinal (GI) absorption is included in Table 3.

According to the ’rule of five’ of drug-likeliness, a good drug candidate for consideration in pre-clinical studies should have a molecular weight (MW) ≤ 500 Da, number of rotatable bonds ≤ 10, number of hydrogen bond acceptors ≤ 10, number of hydrogen bond donors ≤ 5, and a log P_o/w_ value ≤ 5 [45,48]. As shown in Table 3, all the screened phytochemicals fulfill the criteria of drug-likeliness. Similarly, the newly designed heterodimers also conform to these criteria, with only the exception of molecular weight, which was ≤600 Da. The rule associated with MW may certainly limit CNS penetration but has generally proven to be a less rigid requirement for oral drug-likeliness, with a progressive increase in MW over the last decade, with some FDA approved drugs approaching 1000 Da [48]. All the screened phytochemicals have relatively high GI absorption due to smaller size, while the newly designed compounds have either relatively high or low predicted GI absorption, but none were predicted as very low or with no GI absorbance at all, consistent with useful drug-likeliness.

### 3.4. Toxicity Prediction

The pharmacodynamic (toxicological) properties of the phytochemicals and designed heterodimers was predicted using the ProTox-II webserver, with results summarized in Table 4. The predicted acute oral toxicity (LD_50_, dose able to kill 50% of the test animals (rodents)) of the phytochemicals ranged from the least toxic naringenin (2000 mg/kg, toxicity Class IV, harmful if swallowed) to huperzine A (5 mg/kg, toxicity Class II, fatal if swallowed). The majority of phytochemicals had LD_50_ predictions within Classes III or IV. For the heterodimers, the predicted toxicity range was from the least toxic berberastine-4C-carvone (LD_50_ of 1000 mg/kg, harmful if swallowed), to the most toxic, naringenin-4C-galantamine, huperzine-4C-galantamine and galantamine-4C-carvone (LD_50_s of 100 mg/kg, toxic if swallowed). The heterodimer with the highest predicted anti-AChE and anti-BuChE activity, huperzine-4C-naringenin, had a predicted LD_50_ of 280 mg/kg (Class III). Noteworthy, was that none of these designed heterodimers were predicted to be in the severely toxic category of fatal if swallowed (Classes I or II). The tested phytochemicals and their heterodimers were also predicted as inactive for induction of hepatotoxicity (Table 4).

### 3.5. Molecular Dynamic Simulations

**Figure 4:**
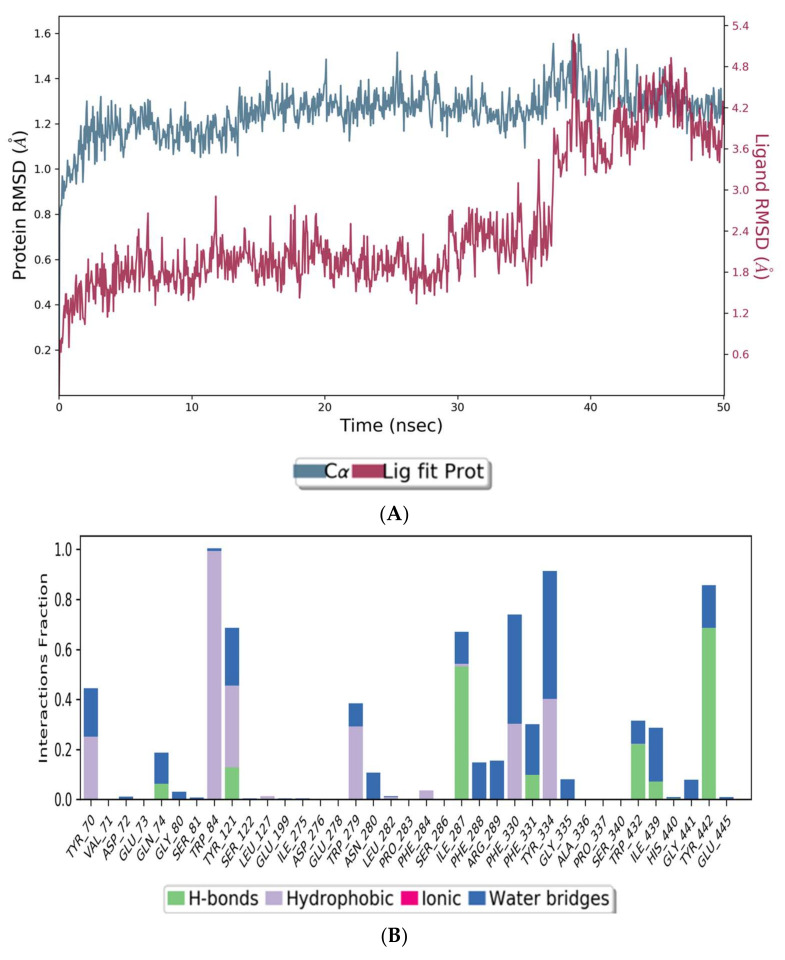

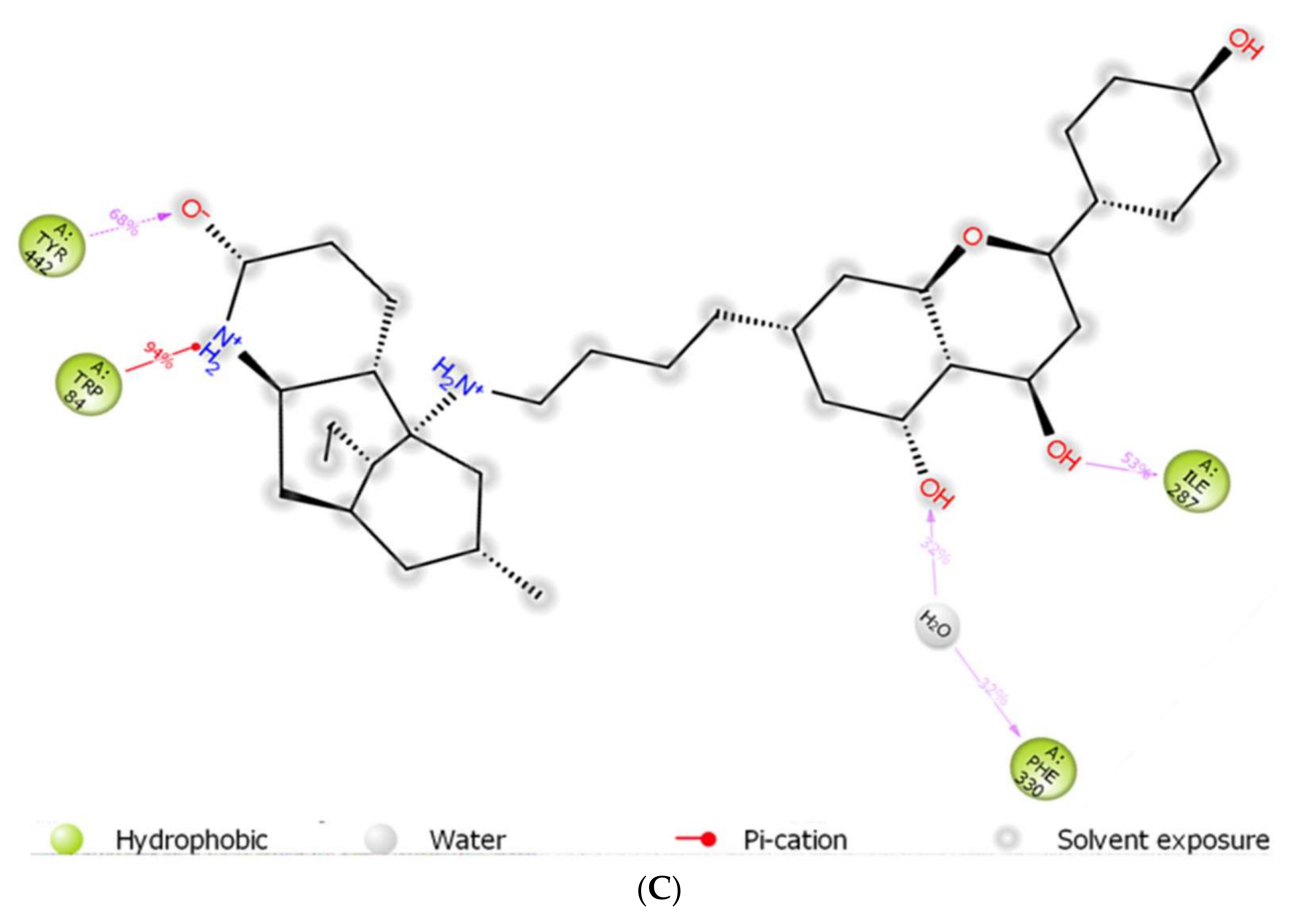
MD simulation studies of huperzine-4C-naranginin in complex with AChE. (**A**) RMSD plot of protein backbone (Cα) and protein conformational change during ligand binding. (**B**) Interaction fraction plot showing different protein residues that interact with the ligand during a 50 ns MD simulation. (**C**) Interaction of ligand atoms with the protein residues that occur for more than 30% of the simulation time.

**Figure 5:**
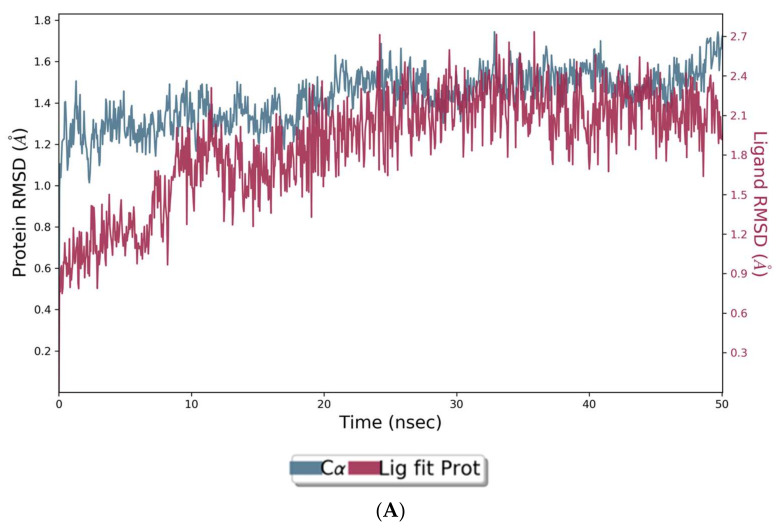

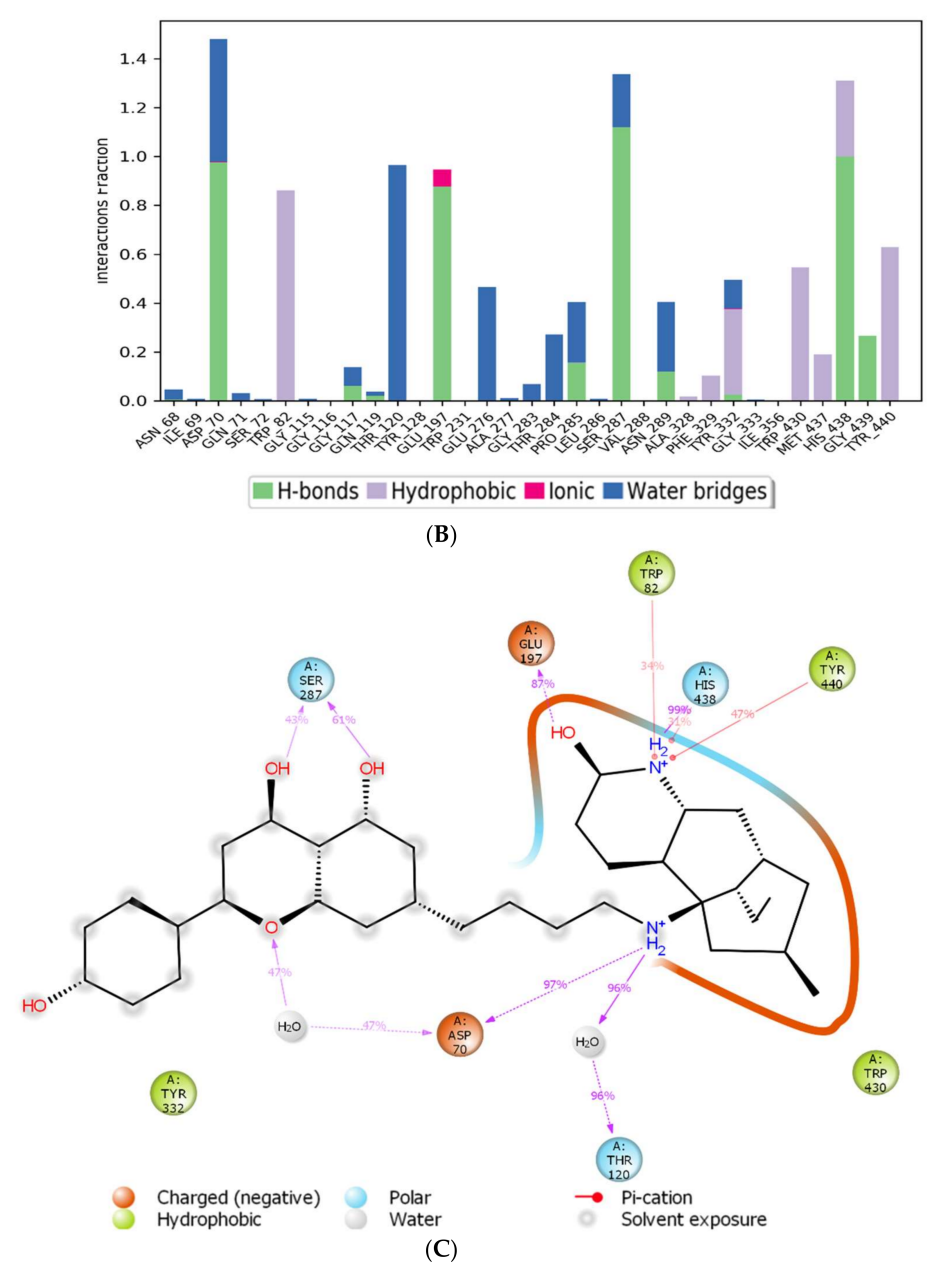
MD simulation studies of huperzine-4C-naranginin in complex with BuChE. (**A**) RMSD plot of protein backbone (Cα) and protein conformational change during ligand binding. (**B**) Interaction fraction plot showing different protein residues that interact with the ligand during a 50 ns MD simulation. (**C**) Interaction of ligand atoms with the protein residues that occur for more than 30% of the simulation time.

The co-crystal structures of AChE and BuChE in complex with huperzine-4C-naringenin docked poses were individually prepared and subjected to molecular dynamic (MD) simulation analysis using the Desmond Molecular Dynamics System [43]. MD simulations were run at NPT for 50 ns and protein-ligand RMSD plots generated for heterodimer binding to AChE (Figure 4A) and BuChE (Figure 5A). The RMSD fingerprints measured the displacement of a selection of atoms over this time period. The interaction of the protein with ligand was also recorded as interaction fraction plots for the heterodimer binding to AChE (Figure 4B) and BuChE (Figure 5B) during the simulation. For binding to AChE, Trp-84 of AChE forms a hydrophobic contact with the ligand for almost 100% of the time, and Tyr-442 and Ile-287 form hydrogen bonds and water bridges for almost 80% of the simulation time. Similarly, Phe-330 and Tyr-334 make hydrophobic contacts and water bridges for 80–90% of the simulation time. Additionally, Tyr-70, Glu-74, Tyr-121, Trp-279, Phe-288, Arg-289, Phe-331, Trp-432, and Ile-439 are other amino acids important for stabilizing the protein-ligand complex (Figure 4B). Figure 4C shows a schematic diagram of several of the major amino acids of AChE involved in the interaction with the huperzine-4C-naranginin heterodimer.

The assessment of the interacting amino acids between BuChE and huperzine-4C-naranginin (Figure 5B,C) shows that Asp-70, Glu-197, Ser-287, and His-438 make hydrogen bonds, and Thr-120 forms a water bridge mediated hydrogen bond with the ligand for 100% of the simulation time. In addition, Trp-82, Tyr-332, Trp-430, and Tyr-440 form hydrophobic interactions with the ligand for 40–90% of the simulation time.

## 4. Discussion

The transient inhibition of AChE via a ChEI monotherapy remains the primary strategy to combat the cholinergic signaling deficit that contributes to the cognitive decline in AD. However, there has been a recent paradigm shift from a single drug, single target approach to the production of drugs with improved efficacy that also simultaneously address multiple components of disease etiology [27,32,33,49,50,51]. For AD, this can reflect the targeting of each of the two drug binding sites available for AChE, the PAS and the CS, as this produces efficacious cholinesterase inhibition and can confer additional treatment benefits including inhibition of Aβ aggregation and modulation of N-methyl-D-aspartic acid (NMDA) receptors [27,32,33,34,35].

With such a dual-binding site strategy in mind, utilization of in silico approaches can streamline the number of drugs selected for subsequent in vitro and in vivo pharmacological evaluation. Hence, herein, phytochemicals with known anti-AChE activity were selected based on an improved (theoretical) binding affinity to AChE than the FDA approved phytochemical drug, galantamine. These phytochemicals were then used to design heterodimers with either a four- or five-carbon (alkyl) spacer, and they were then ranked according to their potency as inhibitors of AChE and BuChE via molecular docking.

The heterodimers were compared directly with the binding affinities of the synthetic drug, donepezil, since donepezil is a potent FDA approved ChEIs and is also capable of binding both the PAS and CS of AChE [3,4,26,52]. However, a limitation of this molecular docking approach is that it only provides a snapshot of the ligand to protein interaction, whereas in nature, this is a dynamic process. Hence, to better understand ligand-protein interaction, molecular dynamic simulations were also performed.

A huperzine-4C-naringenin heterodimer was the most potent theoretical AChE and BuChE inhibitor, as this heterodimer was capable of simultaneous binding to the PAS and the CS of AChE to block enzymatic function. The newly designed ligand was a markedly more potent inhibitor of cholinesterases than galantamine or donepezil. A useful ChEI in part comprised of huperzine A is not surprising since this alkaloid, isolated from the Chinese medicinal herb *Huperzia serrata*, is a traditional Chinese medicine employed for the treatment of cognitive decline [53]. Huperzine A is a recognized efficacious and reversible ChEI and one that displays useful bioavailability and blood-brain barrier penetrance [19,20,21,53,54]. However, huperzine is a relatively impotent BuChE inhibitor by comparison to the FDA approved ChEIs [53]. Furthermore, huperzine homodimers lack potency as ChEIs [54], although this can be improved when coupled to tacrine as a heterodimer [33]. Thus, to better exploit the biological efficacy and safety of huperzine, coupling with a suitable secondary ligand such as naringenin provides both a potent AChE and a potentially powerful BuChE inhibitor (refer to Table 2).

Naringenin is a flavonoid from the flavanones subclass, present in several citrus fruits including lemon, grapefruit, and orange. Naringenin has a number of described biological effects and health benefits, including antioxidant, anti-inflammatory, antibacterial, antiviral, and antitumor activities, as well as hepato- and cardioprotective effects and improvements to metabolic syndrome [55,56]. Most of the studies of naringenin are confined to in vitro characterizations; however, more recently, human clinical trials to establish its health benefits have also been undertaken [56].

Naringenin, purified from *Citrus junos*, is an AChE inhibitor and can ameliorate scopolamine-induced amnesia in mice [57]. Similarly, cholinergic and memory function in type-2 diabetic rats were improved after naringenin treatment, and it was attributed to ChEI and antioxidant activities [58]. Naringenin also inhibited AChE and attenuated behavioral changes in a mice model of social defeat stress [59]. More recently, naringenin, purified from *Drynariae Rhizome*, a traditional Chinese medicine, displayed an AChE IC_50_ of approximately 4 µM [60], consistent with a useful and relatively potent ChEI activity. However, utilization of naringenin as a component of a bioactive ChEI dimer has not been reported before now. Naringenin not only provides the tail group for the most potent AChE and BuChE inhibitor (when combined with huperzine), it is also the next most potent ChEI when arranged as the head group of a dimer with galantamine (refer to Table 2).

Carvone was a component of eight of the eleven heterodimers, a reflection of its relatively low molecular weight (≈150 Da), facilitating a combined heterodimeric molecular weight of less than 600 Da (Table 2). Carvone is a monoterpenoid present in several edible herbs, including spearmint and dill, and is a constituent of mint-based essential oils. Alone, carvone is a relatively poor AChE inhibitor (IC_50_ = 5.56 mM) [61], but our data suggest that it provides a useful phytochemical building block for the production of potent heterodimers.

Yohimbine, an indole alkaloid derived from the bark of the yohimbe tree (*Pausinystalia yohimbe*), has documented pharmacological properties including acting as an α2-adrenoceptor antagonist and is used clinically, primarily to treat male impotence [62]. Although yohimbine has measurable BuChE inhibition in vitro, it is a poor (electric eel) AChE inhibitor [63], although this could be enhanced if coupled to carvone as a heterodimer (Table 2).

Sanguinarine is a benzo[*c*]phenanthridine alkaloid isolated from botanical sources including the root of *Sanguinaria canadensis* (bloodroot). Sanguinarine has purported anticancer activities [64] but may also be carcinogenic itself [65]. Sanguinarine has relatively potent anti-AChE activity with IC_50_ values calculated at approximately 5.6 µM by a high-performance liquid chromatography (HPLC) assay [66] and as low as 0.8 µM via a kinetic assay (of human AChE) [67]; values in keeping with a theoretically high affinity for human AChE (Table 1). By comparison, inhibition of BuChE is weaker, with sanguinarine 14 times less potent an inhibitor of human BuChE than human AChE [67]. However, the generation of a sanguinarine-4C-carvone heterodimer produced a ligand that is potentially a potent inhibitor of BuChE as well as AChE (Table 2).

Berberine, a natural isoquinoline alkaloid often isolated from the Chinese herb *Rhizoma coptidis*, displays a plethora of biologically active properties and similar to sanguinarine, can also induce cytotoxicity [68]. Berberine displays relatively potent anti-AChE binding and inhibitory activity, with IC_50_ values of approximately 0.3–3 µM, in line with theoretical Kd values [29,37,51,66,67,69,70,71]. In comparison, berberine is a weaker inhibitor of BuChE with IC_50_ values in the range of 3–18 µM (for equine BuChE) [69,70]. From our study (Table 2), the potency of berberine as a ChEI was dramatically increased as a heterodimer, in keeping with other computational and in vitro studies of berberine derivatives [29,70].

Chelerythrine is a widely dispersed plant benzo[*c*]phenanthridine alkaloid (Table 1), with a range of biological activities including anti-inflammatory and anti-proliferative (anticancer) effects [71,72]. Chelerythrine is a promising dual cholinesterase inhibitor (IC_50_s of 1.54 µM and 10.34 µM for human AChE and BuChE, respectively) that is also able to inhibit Aβ aggregation [37]. The relative potency of chelerythrine as an AChE inhibitor (affinity of −9.4 kcal/mol, Table 1) could theoretically be increased by 26% as a heterodimer when coupled with carvone (affinity of −11.8 kcal/mol, Table 2).

Berberastine is a plant alkaloid, structurally similar to berberine but hydroxylated (Table 1) and was proposed to be a potent heterodimeric inhibitor of AChE if coupled with either pyrimidine or tacrine via a three- or five-carbon spacer [29]. Coupling of berberastine with carvone (via a 4-carbon spacer) produces a heterodimer, but now with both potent AChE and BuChE inhibitory capabilities (Table 2).

Plant indole alkaloids such as akuammicine display diverse pharmacological activities [73]. Although akuammicine is only a weak AChE inhibitor (IC_50_ = 221 µM, electric eel enzyme) [74], heterodimeric coupling to carvone produced a relatively strong ChEI with near equipotency to inhibit AChE and BuChE (Table 2).

Heterodimer efficacy as ChEIs may be limited through drug bioavailability and access to the brain via movement across the blood-brain barrier (BBB). The heterodimers have predicted CNS penetrance via their physiochemical properties including lipophilicity and conform to the ‘rule of five’ of drug-likeliness (Table 3), albeit for some with a relatively high molecular weight, but that may not be deleterious to movement across the BBB [45,48]. The majority of heterodimers also had predicted GI absorption that was relatively high (Table 3), and this is one indicator of useful bioavailability after oral administration [75].

Only phytochemicals were selected as the chemical building blocks for the dimeric ligands based upon their actual or at least perceived low toxicity due to their natural (as opposed to synthetic) origin [22]. The toxicity of the screened phytochemicals and their heterodimers was predicted using the ProTox-II webserver [42]. The predicted rodent LD_50_ was lowest for berberastine-4C-carvone at 1000 mg/kg, with the majority of other heterodimers in the range of 100–300 mg/kg. By comparison, the predicted LD_50_ from ProTox-II for donepezil and galantamine were 505 mg/kg and 85 mg/kg, respectively (Table 4). This predictive tool performs well with the published oral acute toxicity of galantamine hydrobromide to rats, listed at 75 mg/kg [76] but less comparable with the published acute oral toxicity of the synthetic drug, donepezil hydrochloride to female rats, listed at 32.6 mg/kg [77]. Nevertheless, all of these heterodimers were either class III or IV, and without recognized hepatotoxicity, the rationale for the withdrawal of tacrine as a commercial ChEI [78] (Table 4).

### Summary and Study Limitations

In summary, homo- and heterodimeric ligands have recently come to prominence due to their drug efficacy, dual-site binding, and additional beneficial disease-modifying properties [27,28,29,30,31,32,33,79,80]. In this paper, novel heterodimeric ligands have been designed from phytochemical building blocks that display relatively high binding affinities to both AChE and BuChE and low predicted toxicity. Furthermore, the polyphenolic character of these phytochemicals provides a basis of potent antioxidant activity, agents potentially able to mitigate oxidative stress, another characteristic of AD etiology [81].

The advantage of utilizing an in silico approach is that it provides a basis for rapid, unbiased, and systematic pre-clinical screening, and can be performed in a cost-effective way. However, it is important to consider if in silico predictions are borne out with experimental data. The phytochemicals listed in Table 1 displayed theoretically identical or higher binding affinities to AChE than galantamine. In support of this modeling, the phytochemicals with the relatively higher binding affinities, sanguinarine [67], huperzine A [19], chelerythrine [37], and berberine [37,70] all had experimental AChE IC_50_ values below that of the 2.0 µM calculated for galantamine [19], consistent with relatively high inhibitory potency. By contrast, the three weakest performing affinity ligands, naringenin [60], akuammicine [74] and carvone [61] were 2, 11 and 2780 times, respectively, less active AChE inhibitors in vitro than galantamine, indicative that a threshold of affinity modeling of >−8.5 kcal/mol is needed for consideration of potential experimental potency. Furthermore, yohimbine is not a recognized AChE inhibitor [63] and data has yet to be published for beberastine; thus, structural considerations alone are insufficient to guarantee translation to experimental viability.

Similarly, since the heterodimers described in Table 2 are novel entities without characterization in vitro or in vivo, their true efficacy as ChEIs cannot be documented. Nevertheless, the production of heterodimeric ligands, such as huperzine A coupled with tacrine, have improved efficacy when compared with huperzine homodimers [33], and likewise, berberine coupled to 3-methylpyridinium by a 2-carbon spacer improved the ability to inhibit AChE by approximately 8-fold if compared with berberine alone [70]. Hence, optimism remains as to the efficacy of a heterodimeric approach to inhibit AChE and/or BuChE to rival the current FDA approved drugs: donepezil (AChE IC_50_, 10 nM, BuChE IC_50_, 5 µM) [19], galantamine (AChE IC_50_, 2 µM, BuChE IC_50_, 12.6 µM) [19], or rivastigmine (AChE IC_50_, 4 nM, BuChE IC_50_, 13 nM [82].

The heterodimers were designed to conform to the ‘rule of five’ to provide CNS penetrance, and their GI absorption was predicted (Table 3). However, measures of bioavailability or drug half-life for the heterodimers have yet to be established, and these represent critical elements of oral drug design. Donepezil (hydrochloride) has excellent bioavailability of ≈100%, and with a half-life of ≈80 h, it is suitable for single daily dosing [83,84]. Galantamine (hydrobromide) also has high bioavailability (≈90–100%) but a shorter half-life (than donepezil hydrochloride) at ≈6 h [85], and it typically requires twice-daily dosing. Rivastigmine (tartrate) has a relatively low bioavailability of ≈40% and a relatively short half-life of only ≈1 h and requires twice-daily dosing [86]. Hence, to provide next-generation compounds that are clinically useful, heterodimers will also need to display suitable pharmacokinetic and pharmacodynamic properties to match or improve upon these current therapeutic options.

The modeling of the acute oral toxicity of galantamine was comparable with experimental data, but this was not observed for donepezil, with a 15.5-fold over-estimation (vide supra) (Table 4). Therefore, the acute and organ toxicity of the parent heterodimers as well as their metabolic products will require experimental validation.

Collectively, in silico modeling has provided a means to generate novel heterodimeric ChEIs, but results described herein will need to be supported with laboratory compound synthesis and subsequent in vitro cholinesterase inhibitor validation assays before further pharmacokinetic and pharmacodynamic evaluation in vivo to assess their ability to mitigate cognitive decline in models of AD.

## Figures and Tables

**Table 1:** Screening of most potent phytochemicals with anti-acetylcholinesterase activity.

Ligand	PubChem ID	2D Structures	Mol. Wt g/mol	Example Source Plants *	AChE Affinity (kcal/mol)
Sanguinarine	CID_5154	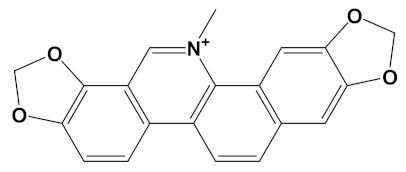	332.3	*S. canadensis*, *A. mexicana*, *B. frutescens*, *C. majus*, *Corydalis spp.*, *E. californica*, *G. flavum*, *M. cordata*	−10.4
Huperzine A	CID_854026	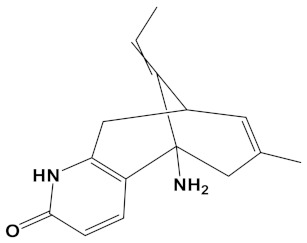	242.32	*Huperzia spp.*, *L. selago*, *L. serratum*	−9.9
Chelerythrine	CID_2703	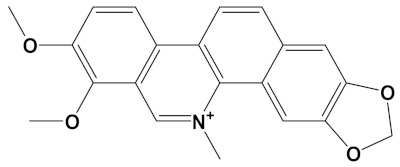	348.4	*A. mexicana*, *Bocconia spp.*, *C. majus*, *E. californica*, *G. flavum*, *S. canadensis*, *Zanthoxylum spp.*	−9.4
Yohimbine	CID_8969	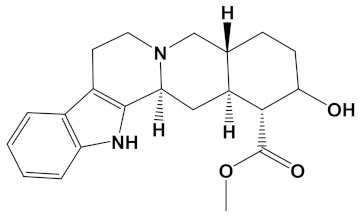	354.4	*A. floribunda*, *P. johimbe*, *Rauvolfia spp.*, *Catharanthus spp.*, *A. quebrachoblanco*	−9.3
Berberine	CID_2353	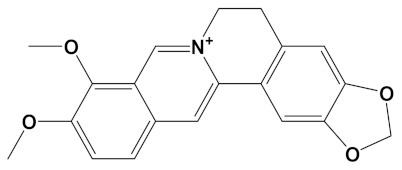	336.4	*B. vulgaris*, *Caulophyllum spp.*, *Coptis spp.*, *Corydalis spp.*, *H. canadensis*, *Mahonia spp.*, *Podophyllum spp.*, *Zanthoxylum spp.*	−9.2
Berberastine	CID_442180	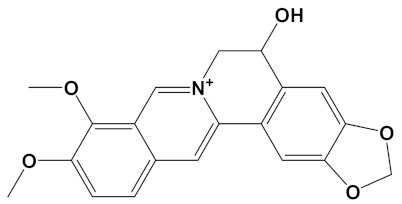	352.4	*Coptis spp.*, *H. canadensis*, *Xanthorhiza simplicissima*	−9.1
Naringenin	CID_932	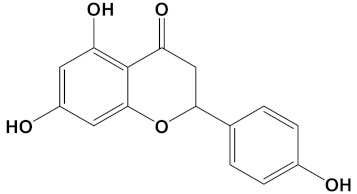	272.25	*Citrus spp.*, *E. globulus*, *A. dracunculus*, *Glycyrrhiza spp.*, *M. pomifera*, *Prunus spp.*, *Salix sp.*, *S. marianum*	−8.5
Akuammicine	CID_10314057	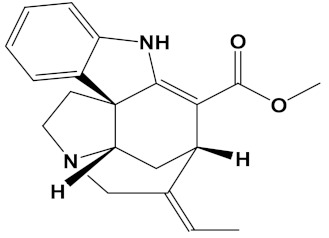	322.4	*A. quebrachoblanco*, *C. roseus*, *V minor*	−8.1
Carvone	CID_7439	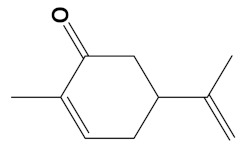	150.22	*Citrus spp.*, *Eucalyptus spp.*, *Mentha spp.*, *Origanum spp.*, *Thymus spp.*, *Teucrium spp.*, *Pycnanthemum spp.*	−7.7
Galantamine	CID_9651	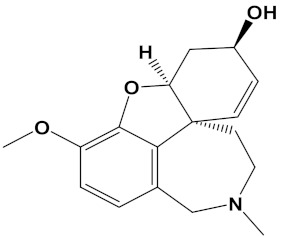	287.35	*G. nivalis*, *H. vittatum*, *L. radiata*, *L. squamigera*, *N. tazetta*, *P. maritimum*	−7.7

* https://phytochem.nal.usda.gov/phytochem/search (accessed on 28 November 2021) [39].

**Table 2:** Dual-binding site heterodimers of potent phytochemicals with anti-cholinesterase activity.

Ligand	Mol. Formula	2D Structures	AChE Affinity (kcal/mol)	BuChE Affinity (kcal/mol)
Huperzine-4C-Naringenin	C_34_H_36_N_2_O_5_	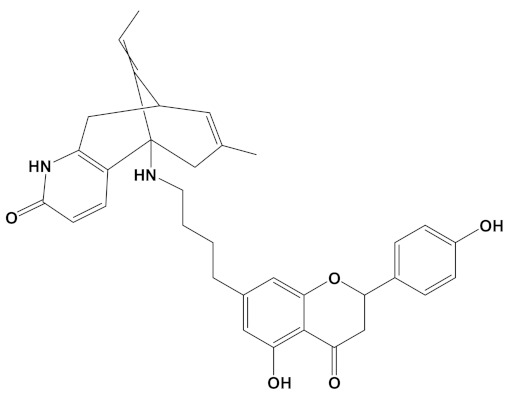	−14.1	−12.3
Naringenin-4C- Galantamine	C_35_H_37_NO_7_	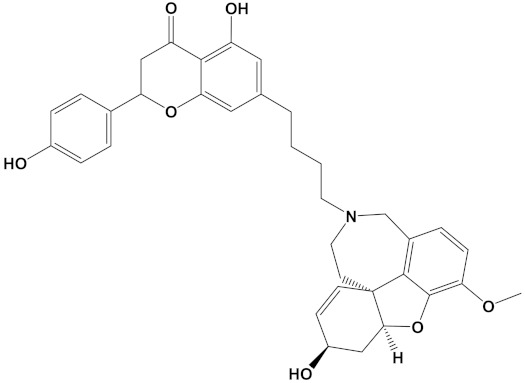	−14.0	−11.9
Huperzine-4C- Galantamine	C_35_H_43_N_3_O_4_	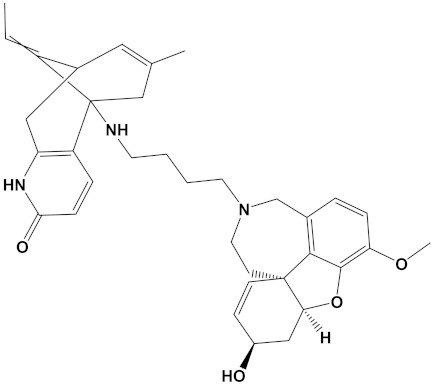	−13.4	−11.4
Huperzine-5C- Carvone	C_30_H_41_N_3_O	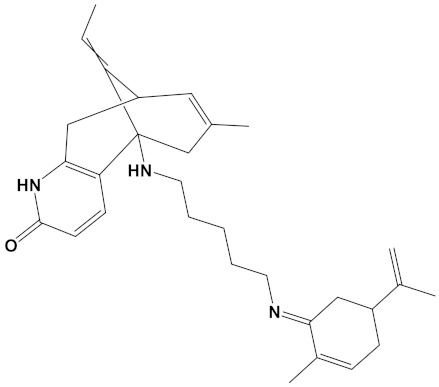	−13.0	−10.0
Yohimbine-5C- Carvone	C_35_H_48_N_4_O_2_	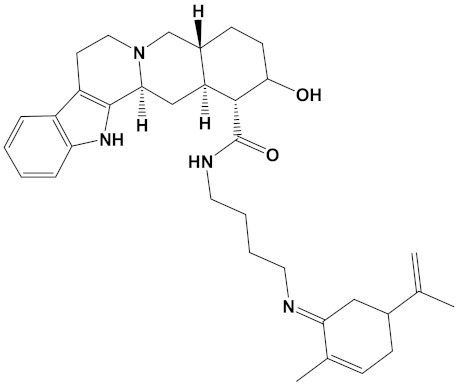	−13.0	−11.6
Galantamine-4C- Carvone	C_30_H_40_N_2_O_3_	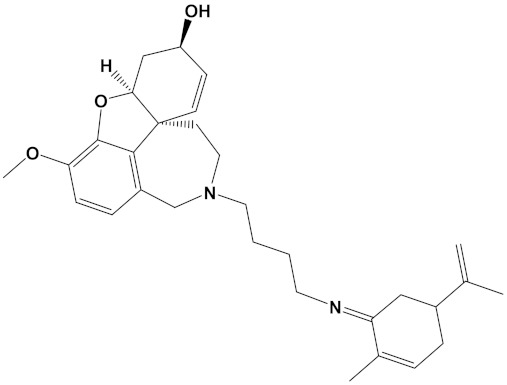	−12.9	−11.0
Sanguinarine-4C- Carvone	C_33_H_34_N_2_O_4_	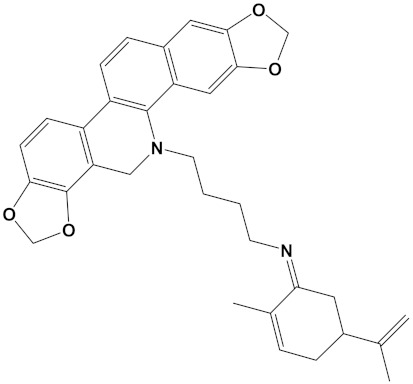	−12.7	−10.4
Berberine-4C- Carvone	C_34_H_39_N_2_O_4_^+^	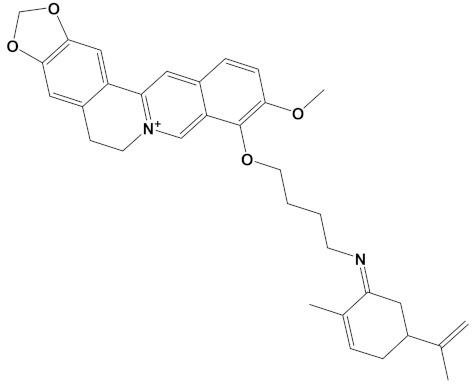	−11.8	−10.3
Chelerythrine-4C-Carvone	C_33_H_37_N_2_O_4_	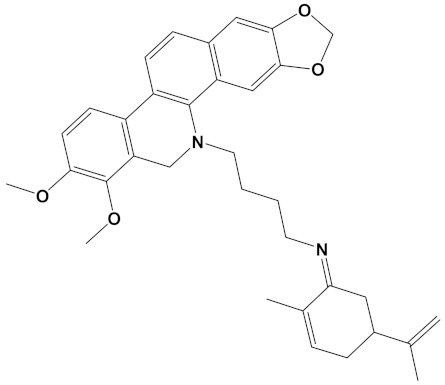	−11.8	−10.3
Berberastine-4C- Carvone	C_33_H_37_N_2_O_5_^+^	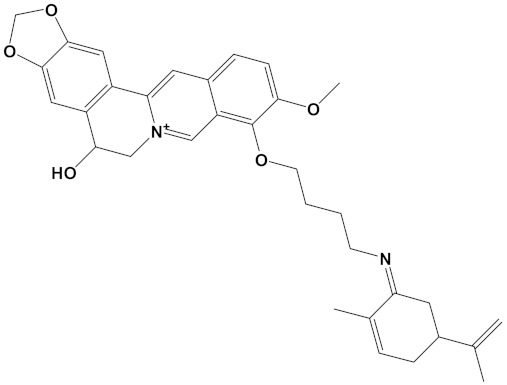	−11.6	−10.4
Akuammicine-4C-Carvone	C_32_H_41_N_3_	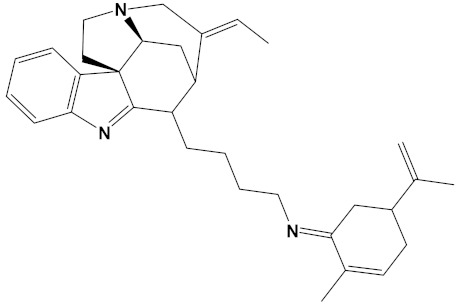	−11.1	−11.6
Donepezil	C_24_H_29_NO_3_	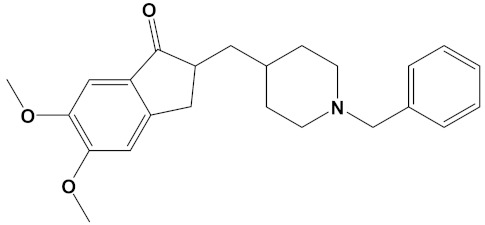	−10.5	−9.8

**Table 3:** Index of ADME properties to predict drug-likeliness of the screened phytochemicals.

Ligand	Mol. Wt. g/mol	Rotatable Bonds	H-Bond Acceptors	H-Bond Donors	Log P_o/w_	GI Absorption
Galantamine	287.4	1	4	1	1.92	High
Sanguinarine	332.3	0	4	0	2.88	High
Huperzine A	242.3	1	2	2	1.88	High
Chelerythrine	348.4	2	4	0	3.02	High
Yohimbine	354.4	2	4	2	2.48	High
Berberine	336.4	2	4	0	2.53	High
Berberastine	352.4	2	5	1	1.72	High
Naringenin	272.3	1	5	3	1.84	High
Akuammicine	322.4	2	3	1	2.60	High
Carvone	150.2	1	1	0	2.44	High
Donepezil	379.5	6	4	0	4.00	High
Huperzine-4C-Naringenin	552.7	7	6	4	3.20	Low
Naringenin-4C- Galantamine	583.7	7	8	3	2.46	High
Huperzine-4C- Galantamine	569.7	7	6	3	3.33	High
Huperzine-5C-Carvone	459.7	8	3	2	4.38	High
Yohimbine-5C-Carvone	556.8	9	4	3	3.53	High
Galantamine-4C- Carvone	476.6	7	5	1	3.43	High
Sanguinarine-4C- Carvone	522.6	6	5	0	4.46	Low
Berberine-4C-Carvone	525.7	8	5	0	3.79	High
Chelerythrine-4C- Carvone	538.7	8	5	0	4.24	Low
Berberastine-4C- Carvone	541.7	8	6	1	2.99	High
Akuammicine-4C- Carvone	467.3	6	3	0	4.50	High

**Table 4:** Prediction of acute oral and hepatotoxicity of the phytochemicals and designed heterodimers.

Ligand	LD_50_ Predicted in Rodent (mg/kg)	Toxicity Class *	Hepatotoxicity
Galantamine	85	III	Inactive
Sanguinarine	778	IV	Inactive
Huperzine A	5	II	Inactive
Chelerythrine	778	IV	Inactive
Yohimbine	300	III	Inactive
Berberine	200	III	Inactive
Berberastine	200	III	Inactive
Naringenin	2000	IV	Inactive
Akuammicine	28	II	Inactive
Carvone	1640	IV	Inactive
Donepezil	505	IV	Inactive
Huperzine-4C-Naringenin	280	III	Inactive
Naringenin-4C-Galantamine	100	III	Inactive
Huperzine-4C-Galantamine	100	III	Inactive
Huperzine-5C-Carvone	150	III	Inactive
Yohimbine-5C-Carvone	300	III	Inactive
Galantamine-4C-Carvone	100	III	Inactive
Sanguinarine-4C-Carvone	296	III	Inactive
Berberine-4C-Carvone	200	III	Inactive
Chelerythrine-4C-Carvone	296	III	Inactive
Berberastine-4C-Carvone	1000	IV	Inactive
Akuammicine-4C-Carvone	325	IV	Inactive

* Class I: fatal if swallowed (LD_50_ ≤ 5). Class II: fatal if swallowed (5 < LD_50_ ≤ 50). Class III: toxic if swallowed (50 < LD_50_ ≤ 300). Class IV: harmful if swallowed (300 < LD_50_ ≤ 2000). Class V: may be harmful if swallowed (2000 < LD_50_ ≤ 5000), Class VI: non-toxic (LD_50_ > 5000).

## Supplementary Materials

The following are available online at https://www.mdpi.com/article/10.3390/cimb44010012/s1, Table S1: List of phytochemicals retrieved from Duke’s Phytochemical and Ethnobotanical databases [39] with reported anti-acetylcholinesterase activity.
