# Supplementary material for: In Silico Design of Dual-Binding Site Anti-Cholinesterase Phytochemical Heterodimers as Treatment Options for Alzheimer’s Disease
Source: Curr Issues Mol Biol. 2021 Dec 29;44(1):152–75. doi: 10.3390/cimb44010012 (PMC8929005; doi:10.3390/cimb44010012)
Supplement: Supplementary file 1 [file cimb-44-00012-s001.zip › cimb-1508384-supplementary.pdf]

Table S1. List of Phytochemicals retrieved from Dr. Duke's Phytochemical and Ethnobotanical databases [39] with reported anti-acetylcholinesterase activity.

	Phytochemical		Phytochemical
1	(-)-Borneol	22	γ-Terpinene
2	(-)-Menthol	23	Huperzine-A
3	(-)-Menthone	24	Isomenthol
4	(-)-Piperitone	25	Isomenthone
5	(-)-Terpinen-4-Ol	26	Isopulegol
6	(+)-Menthol	27	L-Carvone
7	(+)-Piperitenone-Oxide	28	Limonene
8	(+)-Pulegone	29	Linalool
9	(+)-Terpinen-4-Ol	30	L-Limonene
10	1,8-Cineole	31	L-Menthol
11	Akuammicine	32	Menthol
12	Akuammidine	33	Menthone
13	α-Terpinene	34	Naringenin
14	Berberastine	35	P-Cymene
15	Berberine	36	Piperitenone
16	Carvone	37	Pulegone
17	Chelerythrine	38	Sanguinarine
18	D-Carvone	39	Terpinen-4-Ol
19	D-Limonene	40	Viridiflorol
20	Elemol	41	Yohimbine
21	Galantamine		
